# Factors associated with oral pain and oral health-related productivity loss in the USA, National Health and Nutrition Examination Surveys (NHANES), 2015–2018

**DOI:** 10.1371/journal.pone.0258268

**Published:** 2021-10-11

**Authors:** Muath Aldosari, Suellen da Rocha Mendes, Ahad Aldosari, Abdullah Aldosari, Mauro Henrique Nogueira Guimarães de Abreu

**Affiliations:** 1 Department of Periodontics and Community Dentistry, College of Dentistry at King Saud University, Riyadh, Kingdom of Saudi Arabia; 2 Department of Oral Health Policy and Epidemiology, Harvard School of Dental Medicine, Boston, Massachusetts, United States of America; 3 Graduate Program in Dentistry, Universidade Federal de Minas Gerais, Belo Horizonte, Brazil; 4 College of Dentistry at King Saud University, Riyadh, Kingdom of Saudi Arabia; 5 Department of Oral Medicine and Diagnostic Science, College of Dentistry at King Saud University, Riyadh, Kingdom of Saudi Arabia; 6 Department of Community and Preventive Dentistry, Universidade Federal de Minas Gerais, Belo Horizonte, State of Minas Gerais, Brazil; Auburn University, UNITED STATES

## Abstract

**Background:**

Our aim was to identify clinical and sociodemographic factors associated with oral pain and oral health-related productivity loss among US adults.

**Methods:**

We included adults aged ≥30 years who completed the dental examination, had at least one natural tooth, and provided an answer about their oral pain experience or oral health-related productivity loss in the 2015–2018 National Health and Nutrition and Examination Survey (NHANES). We performed descriptive analyses and multivariable binary logistic regression analyses on weighted data.

**Results:**

One out of four adults had oral pain and 4% reported oral health-related productivity loss occasionally or often within the last year of the survey. The odds of oral pain were higher among non-Hispanic black (OR = 1.35; 95%CI = 1.08–1.62) and non-Hispanic Asian individuals (OR = 1.38; 95%CI = 1.07–1.78) compared to non-Hispanic white individuals, and individuals with untreated dental caries (OR = 2.06; 95%CI = 1.72–2.47). The odds for oral health-related productivity loss were 13.85 times higher among individuals who reported oral pain (95%CI = 8.07–23.77), and 2.18 times higher among individuals with untreated dental caries (95%CI = 1.65–2.89). The odds of oral pain and reported oral health-related productivity loss decreased as family income increased.

**Conclusions:**

Factors including non-Hispanic black race/ethnicity, lower socio-economic status, and untreated dental caries are associated with oral pain experience, which increases the odds of oral health-related productivity losses. Identifying factors associated with oral pain and productivity loss will enable clinicians, policymakers, and employers to proactively target and prioritize the higher-risk groups in early interventions and policies.

## Introduction

Chronic oral diseases constitute a range of preventable conditions, primarily dental caries and periodontal disease, which affect more than 3.5 billion people worldwide, especially those living in low- and middle-income countries [1]. Oral diseases are sensitive clinical markers of social disadvantage, and their progression is frequently attributed to a lack of access to oral healthcare, which causes patients considerable pain and discomfort [2,3].

Oral pain is a common clinical symptom of oral disease and has a major influence on vital oral functions, self-esteem, quality of life, and overall health and well-being [2]. Oral pain is also an indicator of severe disease that has been neglected, which can eventually lead to higher treatment costs for the government, health insurance companies, and individuals. The cost of oral pain is not only limited to the direct cost of treatment, but also includes the reduced productive capacity of individuals, with frequent absenteeism at school and work amounting to 33% of the indirect costs of dental care [2,4,5].

Oral problems account for 9–27% of sickness-related absences and 28–50% of presenteeism (reduction of productive capacity in the workplace) among adults, with oral pain being one of the most common reasons [5]. Poor oral health has also been associated with poor academic performance and absenteeism among students at a Brazilian university [6]. Similarly, data from a Canadian survey estimated that over 40 million hours (3.5 hours/person) are potentially lost annually due to dental problems and treatment, with subsequent productivity losses amounting to over $1 billion [7].

In the United States, approximately 2.1 million Emergency Department (ED) visits each year are related to oral pain [8]. Despite the high costs associated with these preventable visits, very limited dental care can be provided in the ED setting. However, many patients still choose the ED as their primary dental care facility, particularly individuals from low-income and minority groups with limited access to professional dental care and insurance through Medicaid [9,10].

Oral pain has been associated with a variety of factors which may lead to adverse psychosocial consequences [11]. However, little is known about the sociodemographic factors associated with oral pain and oral health-related productivity loss among US adults, specially, since some states have expanded Medicaid to include adult dental benefits beyond emergency services [12,13]. Moreover, both oral pain and oral health-related productivity loss are public health concerns, due to their influence on adults’ quality of life and costs to the health care system. Hence, the aim of this study was to identify the clinical and sociodemographic factors associated with oral pain and productivity loss among US adults evaluated in the 2015–2016 and 2017–2018 cycles of the National Health and Nutrition Examination Survey (NHANES).

## Materials and methods

### Study design

We used data from the 2015–2016 and 2017–2018 cycles of the NHANES. This cross-sectional annual survey is conducted by the National Center for Health Statistics (NCHS), a part of the Centers for Disease Control and Prevention (CDC), and examines a representative sample of the non-institutionalized US population, with aggregate findings published every two years. The NHANES collects comprehensive data through self-reported questionnaires, physical examinations, and laboratory investigations. NHANES is one of the main sources of information on oral health status in the US, and includes questions related to oral pain experience and productivity loss [14,15]. We included all individuals aged ≥30 years who completed the dental examination and at least the first 24-hour dietary recall interview, had at least one natural tooth, and provided an answer about their oral pain experience or oral health-related productivity loss. The pooled cycles included 7,299 participants for oral pain outcomes, and 7,304 participants who provided an answer about oral health-related productivity loss. Examinations had an unweighted response rate of 62.3% for the 2015–2016 cycle and 53.7% for the 2017–2018 cycle. Both datasets received NCHS Research Ethics Review Board (ERB) approval, protocol# 2011–17 and 2018–01. The authors were not involved in the data collection, but only obtained the data from the CDC website. All surveyed individuals provided CDC written consent to have data/samples from their data used in research.

All data were fully anonymized before we accessed it.

### Assessment of oral pain and productivity loss

Oral pain and productivity loss were self-reported by participants aged 30 years or older. We categorized oral pain based on responses to the question, “How often during the last year have you had painful aching anywhere in mouth?” Individuals who reported feeling pain “very often,” “fairly often,” or “occasionally” were grouped into one category, while those who answered “hardly ever” or “never” formed another group. To assess productivity loss, we used the same criteria to categorize participants based on responses to the question, “How often during the last year have you had difficulty doing usual jobs or attending school because of problems with teeth, mouth, or dentures?” This question is one of the three questions adopted from original OHIP-49 and used at these NHANNES surveys [16].

### Dental, sugar consumption, and sociodemographic factors

Dentists who performed the clinical oral health examinations were trained and calibrated to ensure reliable measurements among examiners and throughout cycles. We measured the presence of untreated dental caries and restored teeth among dentate individuals. Untreated dental caries was defined as any untreated dental decay involving the crown or root. Filled teeth were defined as teeth with coronal or root restorations for a carious lesion. We also included non-carious root lesions (erosions/abrasions) and restorations of non-carious root lesions in the assessment.

The self-reported sociodemographic risk factors included age (30–34, 35–49, 50–64, and ≥65 years), sex (male or female), race/ethnicity (non-Hispanic white, non-Hispanic black, Hispanic, non-Hispanic Asian, and other), education level (less than high school, high school, and more than high school), and family income ratio to the federal poverty line (FPL) (<100%, 100%-199%, 200%-399%, >400%). We also included the individual’s daily average of total sugar consumption in grams (gm) using the first 24-h dietary recall data collected in-person. The average total sugar consumption was calculated if participants reported the second dietary interview 3–10 days later by telephone.

### Statistical analysis

Descriptive statistics for oral pain experience were obtained for all risk factors, including sociodemographic factors, teeth with untreated dental caries, and teeth with fillings. We recalibrated the weights to report a nationally representative prevalence and population counts of the pooled NHANES data across two cycles by dividing the sample weights by 2. Taylor linearization methods in the survey procedures were used to estimate the standard error (SE) using the supplied masked variance pseudo-stratum and masked variance pseudo-primary sampling units.

Next, we obtained logistic regression models using the survey commands to estimate the socio-demographic and dental factors associated with the outcomes: oral pain experience and oral health-related productivity loss within the last 12 months. We calculated and reported the crude and adjusted odds ratios (OR) of the outcomes, with the corresponding 95% confidence interval (95% CI). After adjusting for all risk factors in multivariable logistic regression for oral pain, we included oral pain as a predictor in the full model for oral health-related productivity loss. We performed a sensitivity analyses by including edentulous individuals, as well as assessing the association for each survey cycle. Multicollinearity was checked by the variation inflation factor (VIF). Cook’s distances were calculated to assess residuals. Hosmer & Lemeshow tests evaluated the goodness-of-fit of the final models. Our estimates were deemed statistically significant at <0.05, and Stata/MP 16 (StataCorp) was used for all the statistical analyses.

## Results

### Oral pain

One out of 4 adults aged 30 years or older in the US reported experiencing oral pain occasionally or often within the last 12 months, representing approximately 41.3 million individuals (Table 1). The highest prevalence of oral pain was reported among the youngest age group (30–34 years; 29.2%), individuals who identified as non-Hispanic black (33.5%) and other race/ethnicity or multi-racial (35.0%), those who had less than high school education (34.7%), and those with a family income <100% of the FPL (38.1%). Regarding dental factors, the highest prevalence of occasional or frequent oral pain within the last year was reported among individuals who had teeth with untreated coronal or root caries (37.5%), those without filled teeth (28.5%), and those with neither non-carious root lesions nor restorations (almost 23.9%). The average daily total sugar consumption among adults 30 years or older in the US was 102.4gm.

**Table 1:** Demographics and prevalence of oral pain among adults aged ≥30 years who completed the dental examination and at least the first 24-hour dietary recall interview, had at least one natural tooth, and provided an answer about their oral pain experience in the National Health and Nutrition and Examination Survey (NHANES); 2015–2018.

	Overall number (%)^†^	Hardly ever, or never experienced oral pain within the last year		Occasionally or often had oral pain within the last year	
		Weighted U.S. population N	(%)	Weighted U.S. population N	(%)
**Overall**	7,299 (100.0)	134,644,000	76.5	41,344,000	23.5
**Age categories**					
30–34	796 (11.6)	14,421,000	70.8	5,956,000	29.2
35–49	2,181 (31.3)	41,834,000	75.9	13,277,000	24.1
50–64	2,416 (33.4)	43,560,000	74.2	15,140,000	25.8
>=65	1,906 (23.8)	34,829,000	83.3	6,970,000	16.7
**Sex**					
Male	3,553 (47.6)	65,584,000	78.2	18,235,000	21.8
Female	3,746 (52.4)	69,060,000	74.9	23,108,000	25.1
**Race/Ethnicity**					
Non-Hispanic White	2,536 (65.3)	91,312,000	79.4	23,672,000	20.6
Non-Hispanic Black	1,611 (10.5)	12.334,000	66.5	6,221,000	33.5
Hispanic	1,983 (14.3)	18,689,000	74.4	6,428,000	25.6
Non-Hispanic Asian	872 (5.7)	7,586,000	75.4	2,475,000	24.6
Other, including multi-racial	297 (4.1)	4,724,000	65.0	2,548,000	35.0
**Education levels**					
Less than high school	1,515 (11.5)	13,212,000	65.3	7,004,000	34.7
High school	1,589 (22.4)	28,352,000	72.0	11,030,000	28.0
More than high school	4,188 (66.1)	93,035,000	80.0	23,284,000	20.0
**Family income ratio to FPL** ^1^					
<100% FPL	1,206 (11.8)	11,749,000	61.9	7,250,000	38.1
100–199% FPL	1,703 (18.4)	20,246,000	68.2	9,449,000	31.8
200–399% FPL	1,793 2(7.5)	33,198,000	75.0	11,080,000	25.0
>400% FPL	1,813 (42.3)	58,205,000	85.4	9,957,000	14.6
**Presence of teeth with untreated caries** ^2^					
No	4,852 (72.4)	104,288,000	81.9	23,127,000	18.1
Yes	2,447 (27.6)	30,363,000	62.5	18,216,000	37.5
**Presence of teeth with fillings2**					
No	1,225 (12.1)	15,166,000	71.5	6,035,000	28.5
Yes	6,074 (87.9)	119,478,000	77.2	35,309,000	22.8
**Presence of non-carious root lesions**					
No	5,141 (71.9)	96,512,000	76.3	29,943,000	23.7
Yes	2,156 (28.1)	38,092,000	77.0	11,401,000	23.0
**Presence of non-carious root restoration**					
No	6,987 (95.6)	128,086,000	76.1	40,194,000	23.9
Yes	310 (4.4)	6,518,000	85.0	1,149,000	15.0

^**†**^The sample counts were unweighted while percentages are weighted to account for complex survey design. The weighted population counts are rounded to the nearest 1000.

^1^FPL: Federal poverty level.

^2^Including coronal and root surfaces.

The unadjusted OR indicates that oral pain generally decreases with age, is more prevalent among minorities than non-Hispanic white individuals, and has an inverse relationship with education level and family income (Table 2). After adjusting for sociodemographic and dental confounders, a lower oral pain experience within the last year was reported by adults aged 35–49 (adjusted OR = 0.71; 95% CI = 0.54–0.93) and elderly participants aged ≥65 years (adjusted OR = 0.46; 95% CI = 0.41–0.68) compared to 30-34-year-old participants. Additionally, the higher odds of oral pain among minorities remained among non-Hispanic black individuals, who had 1.35-fold higher odds (95% CI = 1.08–1.62), and non-Hispanic Asians who reported 1.38-fold higher odds (95% CI = 1.07–1.78) of experiencing oral pain than non-Hispanic white individuals. Females also had a higher odds of reporting oral pain compared to their male counterparts (adjusted OR = 1.34; 95% CI = 1.10–1.64). Furthermore, individuals with more than high school education were less likely to experience oral pain (adjusted OR = 0.65; 95% CI = 0.52–0.82) than those with less than high school education. Even after the adjustment, family income and oral pain experience showed an inverse exposure-response relationship. With each additional 10gm of sugar consumed per day, the odds of oral pain increased by 1.03-folds (95% CI = 1.01–1.04). The presence of coronal or root fillings was associated with a lower odds of oral pain experience, but this association dissipated after adjusting for confounders. On the contrary, the presence of teeth with untreated caries was associated with higher odds of oral pain experience, even after controlling for confounders (adjusted OR = 2.06; 95% CI = 1.72–2.47).

**Table 2:** Logistic regression models of associations between oral pain experience last year and individual characteristics among adults aged ≥30 years who completed the dental examination and at least the first 24-hour dietary recall interview, had at least one natural tooth, and provided an answer about their oral pain experience in the National Health and Nutrition and Examination Survey (NHANES); 2015–2018.

Variable	Unadjusted OR^†^ (95%CI) ^‡^	p-value	Adjusted OR (95%CI)	p-value
**Age categories**				
30–34	Ref^§^		Ref	
35–49	**0.77 (0.60–0.98)**	0.038	**0.71 (0.54–0.93)**	0.016
50–64	0.84 (0.61–1.15)	0.271	0.82 (0.61–1.05)	0.207
>=65	**0.48 (0.37–0.63)**	<0.001	**0.46 (0.41–0.68)**	<0.001
**Sex**				
Male	Ref		Ref	
Female	**1.20 (1.02–1.42)**	0.027	**1.34 (1.10–1.64)**	0.005
**Race/Ethnicity**				
Non-Hispanic White	Ref		Ref	
Non-Hispanic Black	**1.95 (1.59–2.38)**	<0.001	**1.35 (1.08–1.62)**	0.005
Hispanic	**1.33 (1.05–1.68)**	0.020	0.86 (0.65–1.01)	0.247
Non-Hispanic Asian	1.26 (0.95–1.67)	0.105	**1.38 (1.07–1.78)**	0.014
Other, including multi-racial	**2.08 (1.24–3.49)**	0.007	1.50 (0.82–2.75)	0.181
**Education Levels**				
Less than high school	Ref		Ref	
High school	**0.73 (0.60–0.90)**	0.004	0.78 (0.60–1.00)	0.052
More than high school	**0.47 (0.39–0.58)**	<0.001	**0.65 (0.52–0.82)**	0.001
**Family income ratio to FPL** ^1^				
<100%FPL	Ref		Ref	
100%-199% FPL	**0.76 (0.65–0.88)**	0.001	0.86 (0.74–1.01)	0.073
200%-399% FPL	**0.54 (0.46–0.63)**	<0.001	**0.72 (0.61–0.84)**	<0.001
400%	**0.28 (0.20–0.38)**	<0.001	**0.44 (0.32–0.60)**	<0.001
**Total sugar consumption (each additional 10gm)**	**1.03 (1.02–1.04)**	<0.001	**1.03 (1.01–1.04)**	0.001
**Presence of teeth with untreated caries** ^2^				
No	Ref		Ref	
Yes	**2.71 (2.29–3.19)**	<0.001	**2.06 (1.72–2.47)**	<0.001
**Presence of teeth with fillings** ^2^				
No	Ref		Ref	
Yes	**0.74 (0.60–0.92)**	0.007	1.24 (0.96–1.60)	0.101
**Presence of non-carious root lesions**				
No	Ref		Ref	
Yes	0.96 (0.76–1.22)	0.759	1.03 (0.78–1.36)	0.839
**Presence of non-carious root restoration**				
No	Ref		Ref	
Yes	**0.56 (0.36–0.87)**	0.012	0.74 (0.43–1.27)	0.267

Adjusted for age, sex, race/ethnicity, education levels, income, total sugar consumption, presence of teeth with untreated caries, teeth with fillings, root non-carious root lesions and restorations.

^†^OR: Odds ratio.

^‡^95% CI: 95% confidence interval.

^§^Ref: Reference group.

^1^Family income ratio to the federal poverty level (<100%, 100–199%, 200–399%, >400%).

^2^Including coronal and root surfaces.

### Oral health-related productivity loss

The national prevalence of productivity loss due to oral health-related factors was estimated to be 4% among adults aged 30 years and older, which represents 7.0 million individuals who occasionally or often experienced difficulty in performing their jobs or attending school within the last year (Table 3). Individuals aged 50–64 years suffered from the most oral health-related productivity loss (4.7%), and females were more affected (4.1%). Moreover, the highest oral health-related productivity loss was reported among racial/ethnic minorities, individuals with less than high school education (10.7%), and those with a family income-to-FPL percentage <100% (12.8%). In addition, oral health-related productivity loss was higher among individuals who had teeth with untreated coronal and root dental caries (9.3%) and those with neither dental restorations (8.6%) nor non-carious root restorations (4.1%).

**Table 3:** Demographics and prevalence of oral related-productivity loss among adults aged ≥30 years who completed the dental examination and at least the first 24-hour dietary recall interview, had at least one natural tooth, and provided an answer about their oral-related productivity loss in the National Health and Nutrition and Examination Survey (NHANES); 2015–2018.

	Overall number (%)^†^	Hardly ever, or never experienced oral-related difficulty doing usual jobs or attending school within the last year		Occasionally or often had oral-related difficulty doing usual jobs or attending school within the last year	
		Weighted U.S. population N	(%)	Weighted U.S. population N	(%)
**Overall**	7,304 (100.0)	169,077,000	96.0	6,967,000	4.0
**Age categories**					
30–34	796 (11.6)	19,621,000	96.3	757,000	3.7
35–49	2,181 (31.3)	52,710,000	95.7	2,383,000	4.3
50–64	2,419 (33.4)	55,978,000	95.3	2,707,000	4.7
>=65	1,908 (23.8)	40,768,000	97.5	1,058,000	2.5
**Sex**					
Male	3,556 (47.6)	80,636,000	96.2	3,187,000	3.8
Female	3,748 (52.4)	88,442,000	95.9	3,781,000	4.1
**Race/Ethnicity**					
Non-Hispanic White	2,539 (65.4)	111,844,000	97.2	3,204,000	2.8
Non-Hispanic Black	1,612 (10.5)	17,317,000	93.3	1,245,000	6.7
Hispanic	1,984 (14.3)	23,496,000	93.6	1,605,000	6.4
Non-Hispanic Asian	872 (5.7)	9,617,000	95.6	444,000	4.4
Other, including multi-racial	297 (4.1)	6,803,000	93.6	469,000	6.5
**Education levels**					
Less than high school	1,515 (11.5)	18,033,000	89.3	2,160,000	10.7
High school	1,595 (22.4)	37,048,000	94.1	341,000	5.9
More than high school	4,192 (66.1)	113,929,000	97.9	2,463,000	2.1
**Family income ratio to FPL** ^1^					
<100% FPL	1,206 (11.8)	16,551,000	87.2	2,434,000	12.8
100–199% FPL	1,705 (18.4)	27,884,000	93.9	1,821,000	6.1
200–399% FPL	1,793 (27.5)	42,790,000	96.6	1,488,000	3.4
>400% FPL	1,816 (42.3)	67,630,000	99.1	593,000	0.9
**Presence of teeth with untreated caries** ^2^					
No	4,855 (72.4)	125,035,000	98.1	2,435,000	1.9
Yes	2,449 (27.6)	44,042,000	90.7	4,532,000	9.3
**Presence of teeth with fillings2**					
No	1,227 (12.1)	19,380,000	91.4	1,832,000	8.6
Yes	6,077 (87.9)	149,698,000	96.7	5,136,000	3.3
**Presence of non-carious root lesions**					
No	5,145 (71.9)	121,656,000	96.2	4,853,000	3.8
Yes	2,157 (28.1)	47,382,000	95.7	2,114,000	4.3
**Presence of non-carious root restoration**					
No	6,992 (95.6)	161,487,000	95.9	6,850,442	4.1
Yes	310 (4.4)	7,551,000	98.5	117,024	1.5

^**†**^The sample counts were unweighted while percentages are weighted to account for complex survey design. The weighted population counts are rounded to the nearest 1000.

^1^FPL: Federal poverty level.

^2^Including coronal and root surfaces.

After adjusting for demographic and dental confounders, the logistic regression demonstrated that individuals with oral pain had 13.85-fold higher odds of experiencing difficulty in performing their work or attending school due to oral health-related factors (95% CI = 8.07–23.77) (Table 4). Furthermore, Non-Hispanic Asian individuals had a higher odds of reporting oral-related productivity loss than their non-Hispanic White peers (adjusted OR = 1.88; 95% CI = 1.18–3.00). On the other hand, the odds of oral health-related productivity loss were lower among individuals with more than high school education compared to those with less than high school education (adjusted OR = 0.46; 95% CI = 0.30–0.73). We also found an inverse relationship between the family income-to-FPL percentage and oral health-related productivity loss among those with a family income below the FPL. In addition, the presence of teeth with untreated caries increased the odds of productivity loss (adjusted OR = 2.18; 95% CI = 1.65–2.89). The association between race/ethnicity and oral health-related productivity loss dissipated after adjusting for all the other factors.

**Table 4:** Logistic regression models of associations between oral-related productivity loss last year and individual characteristics among adults aged ≥30 years who completed the dental examination and at least the first 24-hour dietary recall interview, had at least one natural tooth, and provided an answer about oral-related productivity loss in the National Health and Nutrition and Examination Survey (NHANES); 2015–2018.

Variable	Unadjusted OR^†^ (95%CI) ^‡^	p-value	Adjusted OR (95%CI)	p-value
**Had oral pain**				
No	Ref^§^		Ref	
Yes	**22.50 (13.86–36.54)**	<0.001	**13.85 (8.07–23.77)**	<0.001
**Age categories**				
30–34	Ref		Ref	
35–49	1.17(0.76–1.80)	0.454	1.42 (0.92–2.19)	0.105
50–64	1.28 (0.77–2.13)	0.323	1.68 (0.90–3.13)	0.098
>=65	0.67 (0.40–1.14)	0.138	1.48 (0.81–2.68)	0.192
**Sex**				
Male	Ref		Ref	
Female	1.08 (0.73–1.60)	0.687	1.25 (0.77–2.03)	0.356
**Race/Ethnicity**				
Non-Hispanic White	Ref		Ref	
Non-Hispanic Black	**2.51 (1.69–3.72)**	<0.001	1.13 (0.70–1.84)	0.605
Hispanic	**2.38 (1.64–3.48)**	<0.001	1.43 (0.99–2.08)	0.054
Non-Hispanic Asian	1.61 (0.95–2.73)	0.074	**1.88 (1.18–3.00)**	0.010
Other, including multi-racial	**2.41 (1.27–4.57)**	0.009	0.91 (0.37–2.23)	0.839
**Education Levels**				
Less than high school	Ref		Ref	
High school	**0.53 (0.38–0.73)**	<0.001	0.86 (0.55–1.35)	0.500
More than high school	**0.18 (0.13–0.25)**	<0.001	**0.46 (0.30–0.73)**	0.001
**Family income ratio to FPL** ^1^				
<100%FPL	Ref		Ref	
100%-199% FPL	**0.44 (0.30–0.66)**	<0.001	**0.58 (0.38–0.87)**	0.011
200%-399% FPL	**0.24 (0.17–0.33)**	<0.001	**0.47 (0.31–0.71)**	0.001
400%	**0.06 (0.04–0.10)**	<0.001	**0.24 (0.15–0.37)**	<0.001
**Total sugar consumption (each additional 10gm)**	**1.05 (1.02–1.07)**	<0.001	1.02 (0.99–1.05)	0.134
**Presence of teeth with untreated caries** ^2^				
No	Ref		Ref	
Yes	**5.28 (4.01–6.95)**	<0.001	**2.18 (1.65–2.89)**	<0.001
**Presence of teeth with fillings** ^2^				
No	Ref		Ref	
Yes	**0.36 (0.24–0.56)**	<0.001	0.74 (0.41–1.35)	0.320
**Presence of non-carious root lesions**				
No	Ref		Ref	
Yes	1.12 (0.83–1.51)	0.447	0.99 (0.74–1.32)	0.966
**Presence of non-carious root restoration**				
No	Ref		Ref	
Yes	0.37 (0.12–1.11)	0.074	0.82 (0.24–2.78)	0.806

Adjusted for age, sex, race/ethnicity, education levels, income, presence of teeth with untreated caries, teeth with fillings, root non-carious root lesions and restorations.

^†^OR: Odds ratio.

^‡^95% CI: 95% confidence interval.

^§^Ref: Reference group.

^1^Family income ratio to the federal poverty level (<100%, 100–199%, 200–399%, >400%).

^2^Including coronal and root surfaces.

All the associations identified in the final models for both outcomes were maintained in the sensitivity analysis. Analyzing each NHANES cycle separately, some estimates of the smaller samples were slightly different than the overall average (S1–S4 Tables). For both outcomes, all the VIF values were close to 1, indicating the absence of collinearity problems. Moreover, Cook’s distances were lower than 1, indicating that the influential cases did not have an effect in the final model. The goodness of fit of the final models were adequate (p > 0.05).

## Discussion

This study evaluated the socio-demographic and dental factors associated with oral pain and oral health-related productivity loss among adults in the US using the NHANES dataset. Nearly one out of four adults in the US experienced oral pain occasionally or often within a year, and 4% reported oral health-related productivity loss. Non-Hispanic blacks, non-Hispanic Asian and disadvantaged populations had higher odds of oral pain or related productivity loss. We also found that oral pain was the strongest factor associated with productivity loss, among other dental and socioeconomic factors.

The prevalence of oral pain was similar to the range identified in a 2001 review of the literature [17] and to findings from more recent oral surveys in Brazil and South Africa [18,19]. Oral pain is considered a public health problem due to its frequency, impact on daily living, and the feasibility of prevention. In the first oral health report of the surgeon general, oral health was deemed an integral part of general health [11]. In addition, safe and effective measures exist to prevent most oral diseases. Thus, the current challenges in oral pain management include the lack of dental coverage among adults and access to dental services [14,20].

The role of socioeconomic status in oral pain has already been demonstrated in different contexts [17–19]. Social disparities have been also identified historically; most socially vulnerable populations are at high risk of oral disease [11,21], and the barriers to accessing timely dental treatment increase the odds of oral pain [20]. In turn, the presence of untreated dental caries can also increase the odds of oral pain, as it is the most common condition that causes pulpitis [17]. The association between sex and oral pain has been considered controversial in the literature. Some authors did not identify any association, despite others have found that women were more prone to have dental pain [22–24]. The sex differences in the use of dental services and in the self-report of heath conditions could explain the association identified in our study [25,26].

In the US, non-Hispanic black adults have the highest odds of untreated dental caries, in contrast to non-Hispanic white adults [27]. This statistic may partially explain the association between non-Hispanic black adults and oral pain in our study, even after adjusting for confounding factors. Furthermore, studies have indicated that patients visiting the ED due to oral pain disproportionately included black and low-income men, which emphasizes that these populations have less access to preventive and restorative dental treatments. Consequently, these groups reported more oral pain episodes than other ethnic groups [9,10]. The literature on self-reported health among Asians in the United States is scarce. Some evidence from NHANES identified that non-Hispanic asians were more likely to report moderate health than excellent/very good health status [28]. The study explained that immigrants in the United States may adopt unhealthy lifestyle and behaviors, which could be due to the psychological stress and racial discrimination, resulting in worse self-report of health.

Behavioral factors, such as sugar consumption, increase the risk of developing dental caries, and as consequence, oral pain [22]. Elderly individuals in the US reported less oral pain than younger adults, potentially due to the fact that pulpitis is more common among younger patients [17]. Although periodontitis and root caries are more common among older patients, these patients often retain fewer natural teeth due to tooth loss, as a result of the cumulative burden of oral disease [29].

In the present study, 7.0 million adults (4%) reported productivity loss due to oral problems within the last year, with oral pain and untreated caries being the strongest factors. The prevalence of absenteeism from work or school due to dental disease varies in the literature, ranging from 9% in an Australian study to 35.1% in a Canadian study [7,30]. However, many of individuals still go to work and carry out daily activities while experiencing an oral pain episode (presenteeism), which can impair their productivity, promote attention loss, irritability, anxiety, and depression, and increase the risk of work-related accidents [5]. In the US, almost 321 million hours of work and school are lost per year due to dental problems, of which emergency dental treatment accounted for 92.4 million work or school hours [31]. A Canadian study revealed that individuals lose an average of 3.5 working hours each year due to dental problems, which amounts to 40 million hours per year. Oral health-related productivity loss is estimated to cost $30.19 billion annually in the US and Canada [4].

The literature describes the importance of oral health for quality of life, as well as the impact of oral pain on daily activities, especially at work and at school [5–7]. In addition to causing pain, dental diseases can limit patients’ ability to properly chew, taste, swallow, smile, and find a mate. Dental diseases also compromise the psychosocial well-being and influence self-expression, communication, and facial aesthetics, which hinder employment opportunities, especially among disadvantaged individuals [32–34].

Considering the significant association between oral pain and oral health-related productivity loss, we found common socioeconomic factors associated with both outcomes. Although we found that less education and lower income were related to productivity loss, a study by Hayes et al.^7^ found that higher education level and income were associated with greater work absenteeism due to dental diseases and attempts to seek dental treatment [7]. On the contrary, an evaluation of dentate adults from NHANES 2003–2004 found that higher-income individuals had a lower oral health-related quality of life (OHRQoL), which included productivity loss [35].

To tackle the challenges imposed by unmet dental needs, one of the national Healthy People 2030 objectives is to “reduce the proportion of people who can’t get the dental care they need when they need it” [36]. With cost being the most reported barrier to dental care, it is even more difficult to address the unmet dental needs among the most vulnerable adults who have no dental coverage [37,38]. The provision of dental coverage for cost-effective preventive and early treatments for adults and elderly patients through Medicaid and Medicare could potentially reduce the burden on the utilization of ED, reduce productivity losses caused by dental diseases, and save costs in the long run [39].

Although the sampling and measurement methods, including clinical examinations, used to obtain the data used for this analysis are sufficiently valid and rigorous enough to represent the oral health of the US population, the study had some limitations. The cross-sectional survey design of NHANES made it impossible to evaluate temporality. Thus, we cannot validate the assumption that education, income, and dental diseases preceded our outcomes. In addition, the measurements of oral pain experience and oral health-related productivity loss were not quantifiable, and the timeframes for the questions could potentially introduce a recall bias. The recall bias could also lead to differential misclassification of the outcomes among individuals with severe dental diseases and those with mild dental disease. Lastly, the outcomes were measured among individuals aged 30 years or older, which limits our ability to make inferences about younger groups. Another limitation of this study is the findings obtained may not be generalized to other population or settings outside of US. Nevertheless, this is the first study to evaluate oral pain, productivity loss, and associated factors among adults in the US using the NHANES dataset.

## Conclusions

Oral health is an integral part of overall well-being; thus, oral pain can hinder the ability to achieve an optimal quality of life. Identifying factors associated with oral pain and productivity loss will enable clinicians, policymakers, and employers to proactively target and prioritize the higher-risk groups in early interventions and policies. Dental students and dentists should aim to identify the social determinants of oral pain and productivity loss, so they can gain more social competence in recognizing health disparities.

## Supporting information

S1 Table Logistic regression models of associations between oral pain experience last year and individual characteristics among adults aged ≥30 years who completed the dental examination and at least the first 24-hour dietary recall interview, had at least one natural tooth, and provided an answer about their oral pain experience in the National Health and Nutrition and Examination Survey (NHANES); 2015–2016 (N = 3,714). (TIF)

S2 Table Logistic regression models of associations between oral-related productivity loss last year and individual characteristics among adults aged ≥30 years who completed the dental examination and at least the first 24-hour dietary recall interview, had at least one natural tooth, and provided an answer about oral-related productivity loss in the National Health and Nutrition and Examination Survey (NHANES); 2015–2016 (N = 3,718). (TIF)

S3 Table Logistic regression models of associations between oral pain experience last year and individual characteristics among adults aged ≥30 years who completed the dental examination and at least the first 24-hour dietary recall interview, had at least one natural tooth, and provided an answer about their oral pain experience in the National Health and Nutrition and Examination Survey (NHANES); 2017–2018 (N = 3,584). (TIF)

S4 Table Logistic regression models of associations between oral-related productivity loss last year and individual characteristics among adults aged ≥30 years who completed the dental examination and at least the first 24-hour dietary recall interview, had at least one natural tooth, and provided an answer about oral-related productivity loss in the National Health and Nutrition and Examination Survey (NHANES); 2017–2018 (N = 3,586). (TIF)
