# Supplementary figures and images for: Factors associated with oral pain and oral health-related productivity loss in the USA, National Health and Nutrition Examination Surveys (NHANES), 2015–2018
Source: PLoS One. 2021 Oct 11;16(10):e0258268. doi: 10.1371/journal.pone.0258268 (PMC8504739; doi:10.1371/journal.pone.0258268)

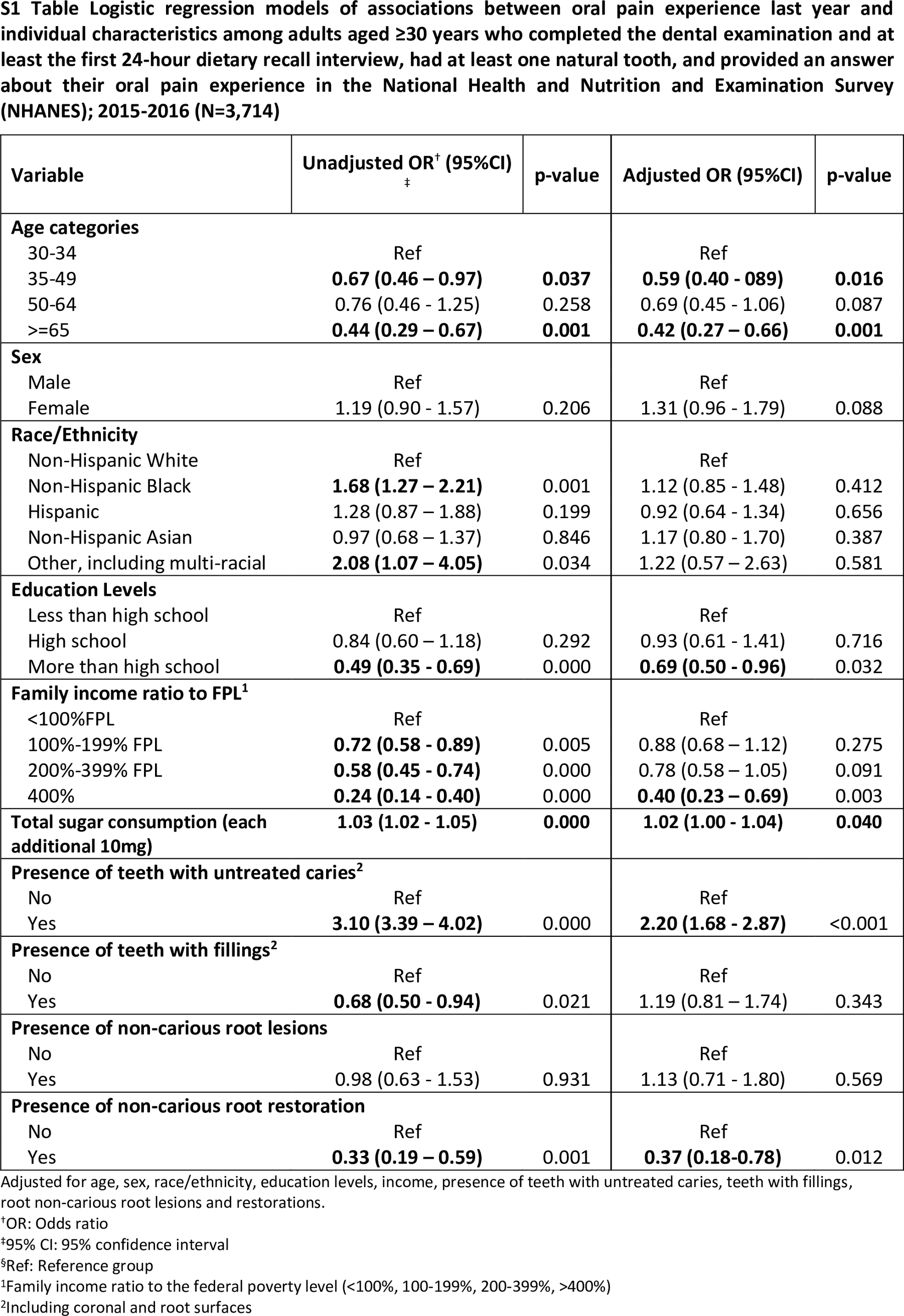

Supplement: S1 Table — (TIF) [file pone.0258268.s001.tif]

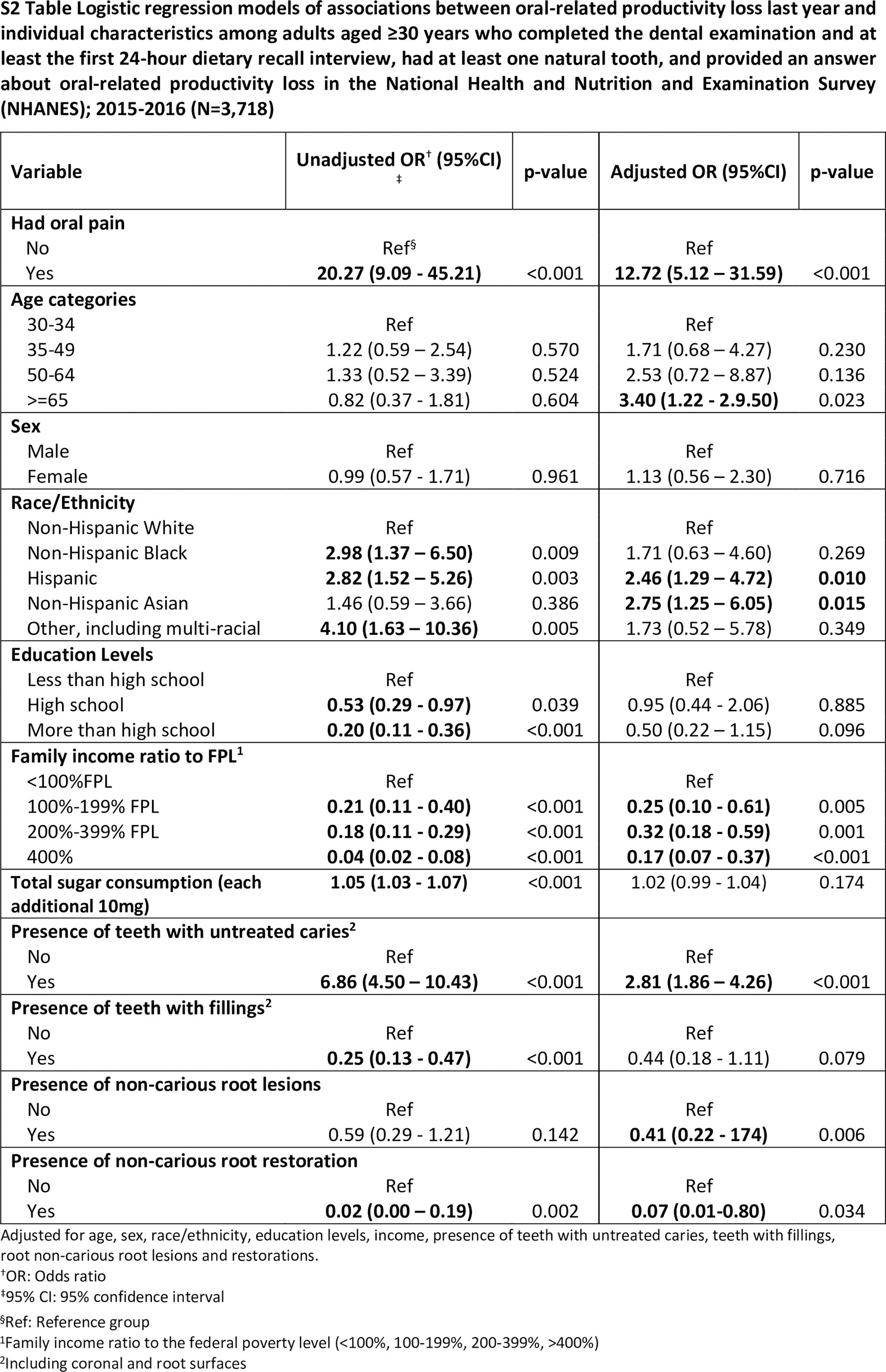

Supplement: S2 Table — (TIF) [file pone.0258268.s002.tif]

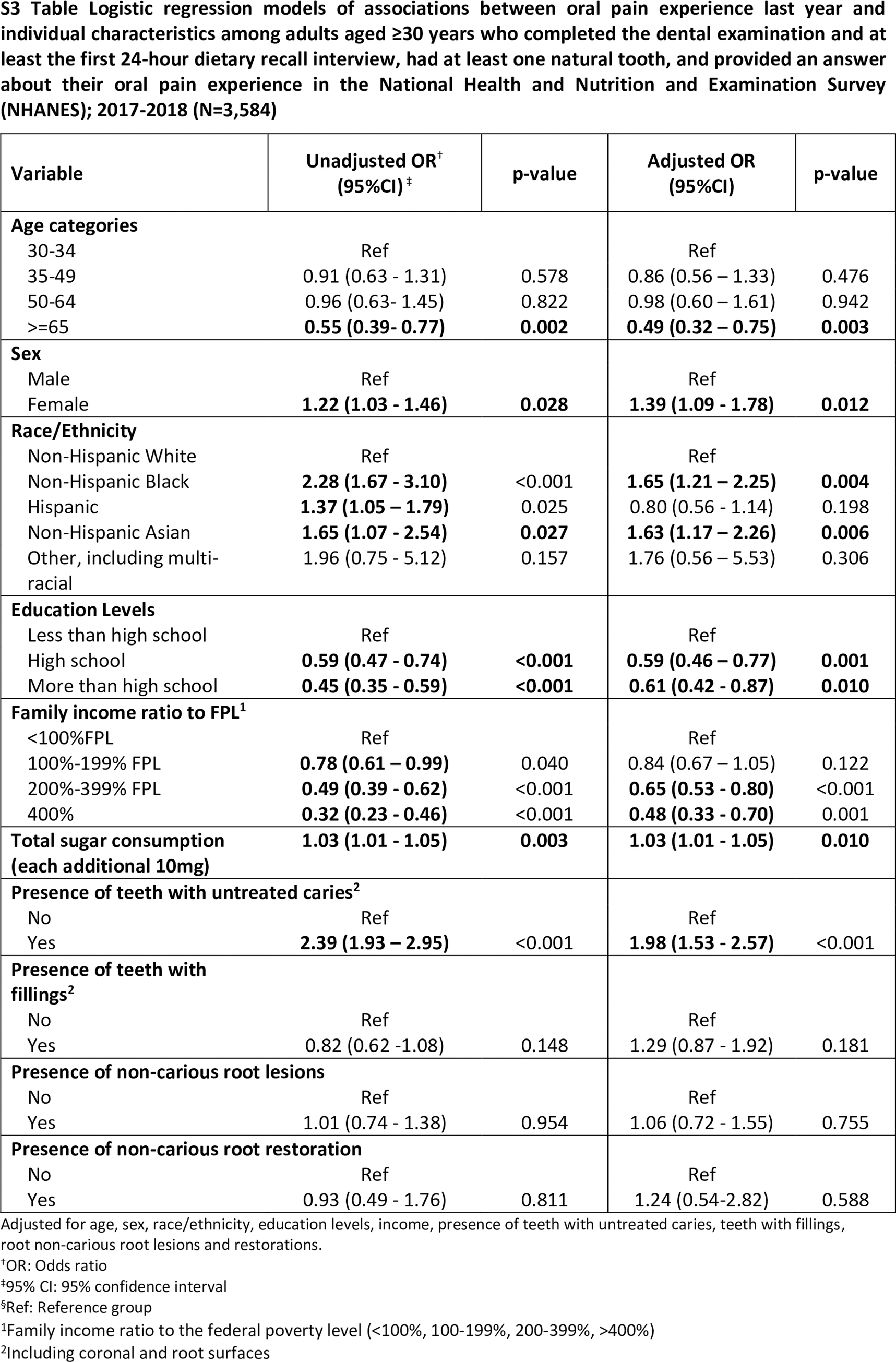

Supplement: S3 Table — (TIF) [file pone.0258268.s003.tif]

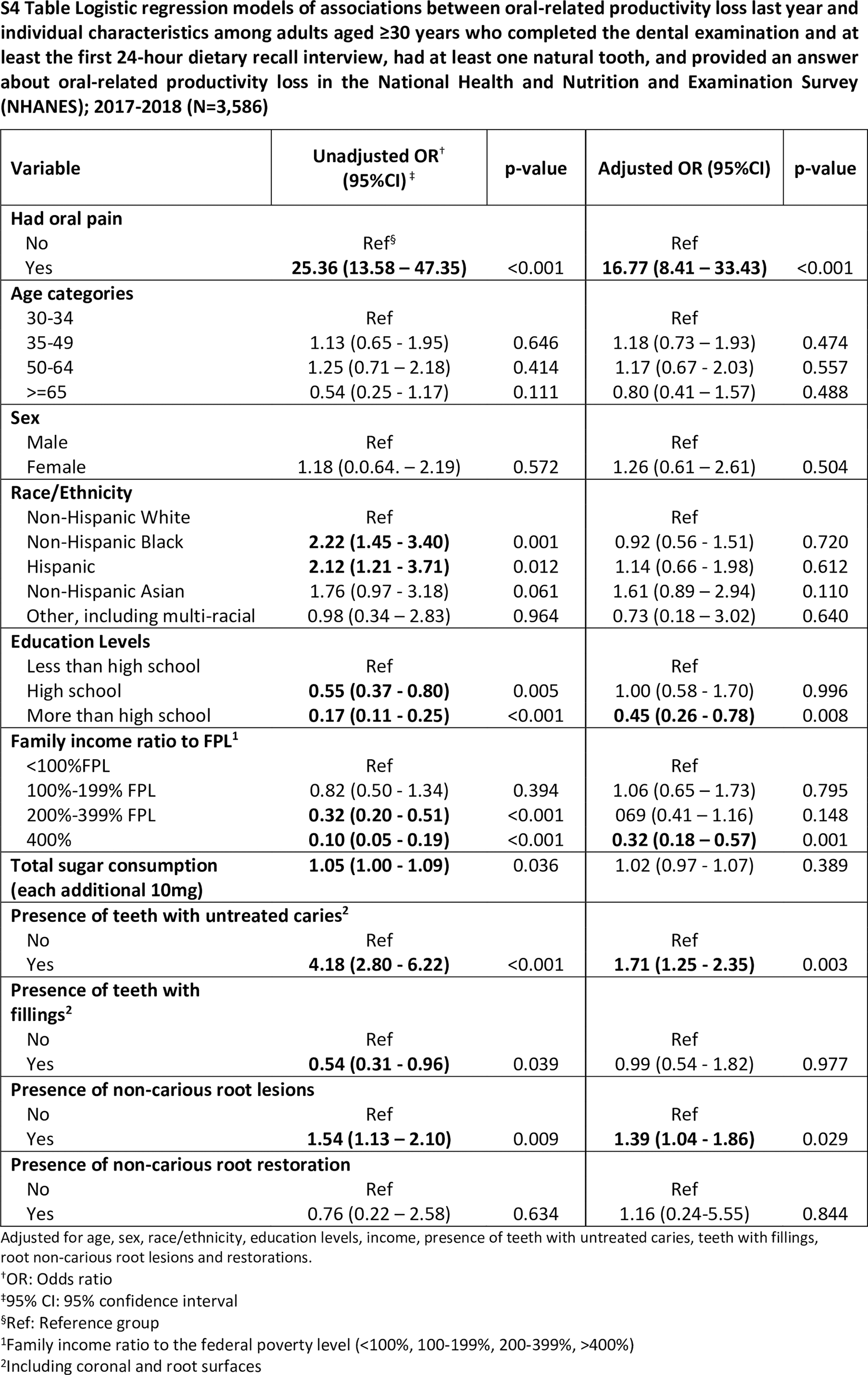

Supplement: S4 Table — (TIF) [file pone.0258268.s004.tif]
